# Dietary supplementation with milk‐derived *Bifidobacterium animalis* subsp*. lactis* relieves colitis‐linked reproductive disorders via gut‐testis axis

**DOI:** 10.1002/imo2.49

**Published:** 2025-01-02

**Authors:** Jingmin Lin, Lingzi Yin, Yueyao Fan, Zhiling Yu, Xin Ma, Xiuqiong Fu, Yong Zhang, Shaojun Tang, Jiali Chen

**Affiliations:** ^1^ Department of Food Science and Engineering, College of Life Science and Technology Jinan University Guangzhou China; ^2^ Bioscience and Biomedical Engineering Thrust, Systems Hub The Hong Kong University of Science and Technology (Guangzhou) Guangzhou China; ^3^ Centre for Cancer and Inflammation Research, School of Chinese Medicine Hong Kong Baptist University Hong Kong China; ^4^ State Key Laboratory of Bioreactor Engineering East China University of Science and Technology Shanghai China; ^5^ Institutes for Systems Genetics, West China Hospital Sichuan University Chengdu China; ^6^ Center for Aging Science, Division of Emerging Interdisciplinary Areas The Hong Kong University of Science and Technology Hong Kong China

**Keywords:** *Akkermansia muciniphila*, *Bifidobacterium animalis* subsp. *lactis*, gut‐testis axis, metabolomics, ulcerative colitis

## Abstract

Rapid urbanization, medication, and modern dietary patterns are the main challenges leading to impaired fertility, with a lack of effective therapies. Emerging evidence suggests that reproductive disorders may be closely associated with intestinal damage or occasionally worsen by the side effects of medications. Therefore, the development of dietary supplements as alternatives is crucial for intestinal‐linked reproductive health. Milk and dairy products are essential in dietary nutrition with great potential functional ingredients. *Bifidobacterium animalis* subsp*. lactis* NJ241 (NJ241), a promising probiotic isolated from naturally fermented bovine milk, remains underexplored. This study aims to investigate the molecular mechanisms of NJ241 on colitis and its associated reproductive disorders. The biomarker microbes and their correlated metabolites were further explored by 16S rRNA sequencing and metabolomics. Hematological analysis and histopathological examination were applied for conjoined identification. Results indicated that NJ241 effectively restored the expression levels of Claudin‐2 and MLCK1, reducing intestinal permeability. Multi‐omics results further revealed that NJ241 may effectively improve gut barrier integrity by increasing the abundance of *Akkermansia muciniphila* and its metabolite trans‐ferulic acid. This effect was accompanied by a reduction in the pro‐inflammatory cytokine IL‐6 in both serum and testicular tissue, mediated through the TLR4 signaling pathway. Consequently, the restoration of microbiota homeostasis and a systemic reduction in inflammation rescued testicular spermatogenesis, which was impaired by colitis. The current findings consistently elucidated the potential molecular mechanism by which NJ241 ameliorates colitis‐linked reproductive disorders through the gut‐testis axis. Additionally, NJ241 demonstrates promise as a probiotic supplement for the development of fortified dairy products and provides strong evidence for the potential reproductive health benefits of naturally fermented bovine milk.

## Highlights

- The maintenance of microbial homeostasis and low‐grade inflammatory ecology could improve testicular spermatogenesis.
- *Bifidobacterium animalis* subsp*. lactis* NJ241 may effectively ameliorate colitis‐linked reproductive disorders through the gut‐testis axis.
- Established the feasibility of *Bifidobacterium animalis* subsp*. lactis* NJ241 as the potential probiotic for male reproduction improvement.
- Provided the prospective application of health benefits of naturally fermented bovine milk.

## Graphical abstract

The illustration of health benefits of *Bifidobacterium animalis* subsp*. lactis* NJ241 isolated from naturally fermented bovine milk. Integrative approaches unraveled that daily supplementation with *Bifidobacterium animalis* subsp. *lactis* NJ241 could markedly suppress colitis‐linked reproductive disorders through the gut‐testis axis. *Bifidobacterium animalis* subsp. *lactis* NJ241 may effectively improve gut barrier disorder by increasing the abundance of *Akkermansia muciniphila* and its metabolite trans‐ferulic acid. Subsequently, the expression levels of pro‐inflammatory cytokine IL‐6 were suppressed in both serum and testicular tissue, which is closely related to the improvement of sperm motility and viability. It unveiled the prospective pharmacological mechanism of fermented milk‐derived probiotic *Bifidobacterium animalis* subsp. *lactis* NJ241 in promoting reproductive health benefits.

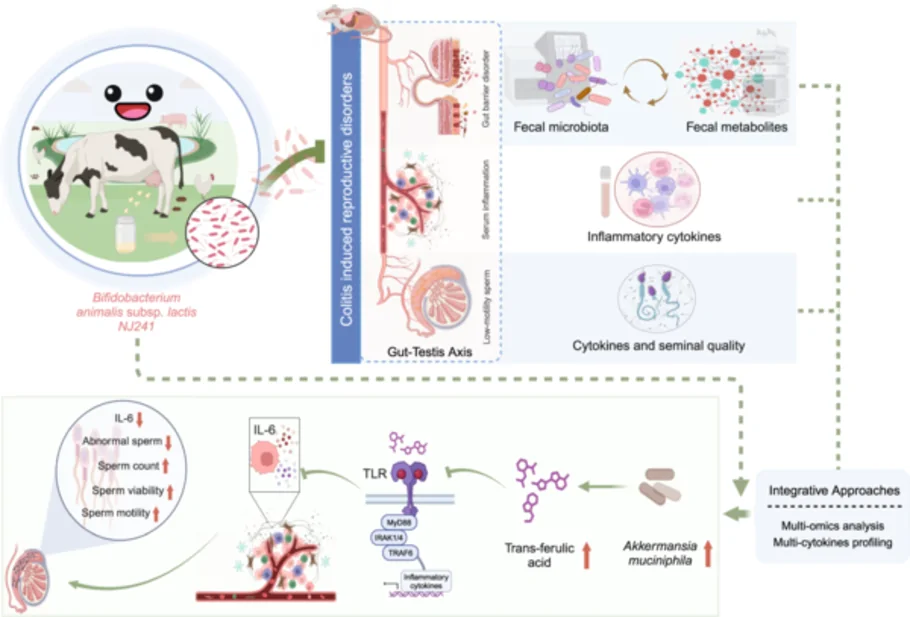

## 1 INTRODUCTION

Ulcerative colitis (UC) is a chronic idiopathic inflammatory bowel disease (IBD) that causes persistent mucosal inflammation in the colon [1]. While IBD can occur at any age, it is most prevalent in young adults of reproductive age, making concerns about the safety of medications and the potential impact of the disease on fertility and pregnancy [2]. Infertility is defined as the inability to conceive after at least 12 months of regular, unprotected sexual intercourse [3]. More attention has been paid to fertility in women with IBD but less to the reproduction function of men with IBD. However, male infertility is significantly more prevalent among patients with IBD compared to the general population [4]. In men with UC, studies have shown a significant decline in sperm quality, including reduced total sperm count, viability, progressive motility, and the proportion of morphologically normal spermatozoa [5]. The health status or balance of the gut microbiota may influence the development and health of the male reproductive system in mammals [6]. With up to 12% of men suffering from infertility as well as sexual dysfunction, the connections between gut health and male fertility demand greater attention [7]. Furthermore, the therapeutic drugs used to treat IBD are known to adversely affect male fertility, highlighting an urgent need for safer and more effective treatments [8].

Although the pathogenesis of UC remains unclear, its development appears to be linked to ecological dysbiosis, a disruption in gut microbial diversity driven by an imbalance between commensal and pathogenic microorganisms [9]. The gut microbiota, often regarded as the second human genome, is critical for maintaining the balance between the host's external and internal environments [10, 11]. UC patients are typically associated with a reduction in beneficial bacteria, such as *Eubacterium rectale* and *Akkermansia*, and an increase in harmful bacteria like *Escherichia coli* [12]. Additionally, emerging evidence suggests that gut microbiota imbalance may affect male fertility [13]. For instance, studies in a sheep model of dietary metabolic disorders revealed that gut dysbiosis reduced the levels of bile acids, disrupting the transport of fat‐soluble vitamin A in the gut‐testis axis and subsequently impairing spermatogenesis in the testis [14].

Probiotics have shown a potential to alleviate UC by restoring gut microbiota balance and suppressing intestinal inflammation [15, 16]. This suggests that reshaping gut microbiota homeostasis and intestinal metabolism could mitigate UC‐induced male reproductive dysfunction. Exploring gut microbiota modulation and its metabolites may be crucial to understanding the molecular mechanism of UC and its systemic effects. While pharmacological treatments remain the standard approach for UC, they are often associated with various adverse effects. This underscores the need of exploring natural diet therapies or supplements, which may offer safer and effective options for UC management.

Milk, a natural secretion of mammals, provides a nearly complete nutritional profile for humans. Studies suggest that fortifying human milk with bovine milk could be an effective nutritional supplement for infants [17]. Additionally, milk has been linked to improved gut health due to its functional bioactive ingredients [18]. Identifying these bioactive substances is essential for advancing milk‐related industrial applications. One promising candidate is *Bifidobacterium animalis* subsp*. lactis* NJ241 (NJ241), a potential probiotic isolated from naturally fermented bovine milk. Although research data on NJ241 are limited, a report suggests that *Bifidobacterium* may be applied as an effective adjuvant treatment for UC [19]. *Bifidobacterium* is one of the main and vital microorganisms to inhabits the intestinal tracts of humans and animals [20].

Currently, the specific role of the gut microbiota in the gut‐blood‐testis axis remains unknown. This study aims to investigate the molecular mechanisms of NJ241 on colitis and its associated reproductive disorders through a conjoined analysis of the intestinal flora and fecal differential metabolites. It may deliver great significance to develop effective dietary supplements as the medical alternative for intestinal‐linked reproductive health.

## 2 RESULTS

### NJ241 alleviates colonic inflammation and tissue damage in ulcerative colitis

**FIGURE 1:**
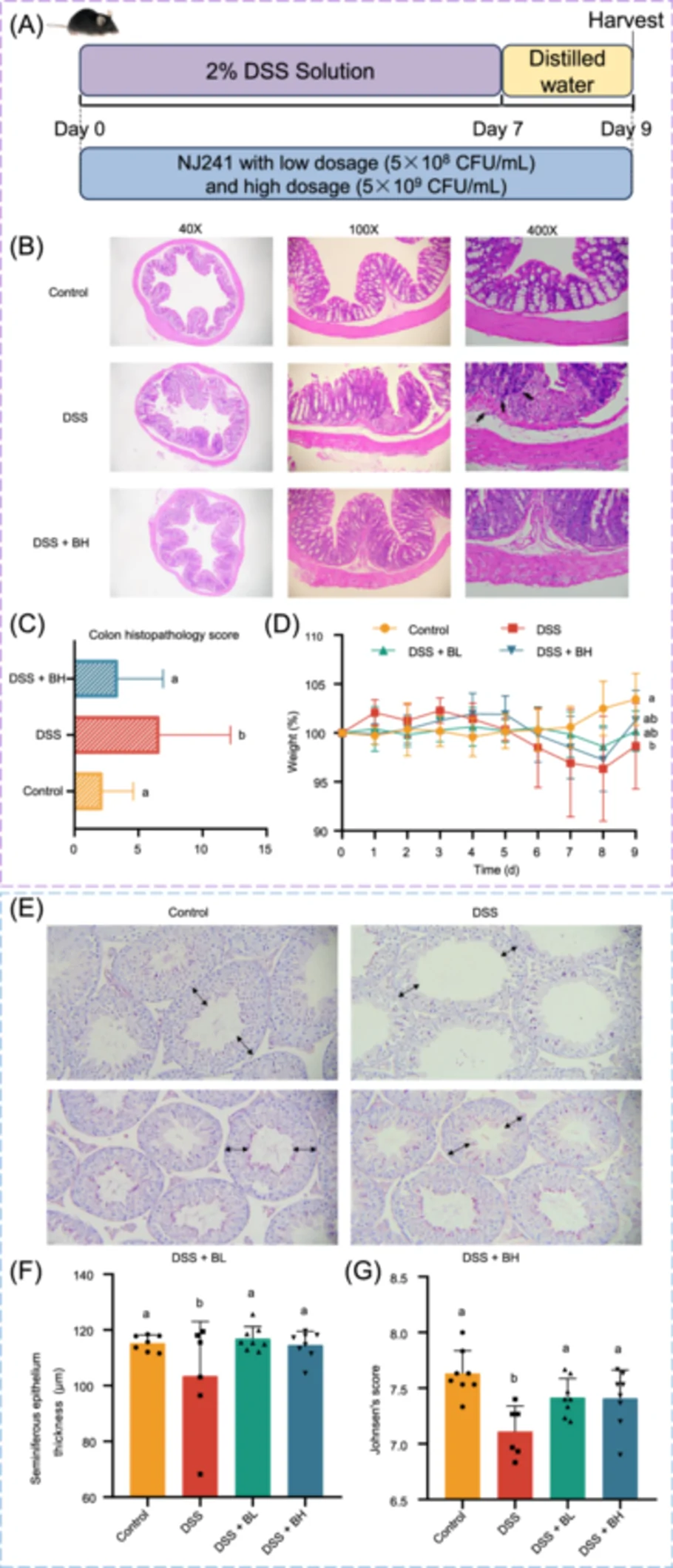
Effects of *Bifidobacterium animalis* subsp. *lactis* NJ241 on ulcerative colitis (UC)‐induced male reproductive damage symptoms in mice. (A) Experimental timeline of the mouse model of colitis. (B) Histological analysis. (C) Daily body weight changes throughout the entire duration of the study. (D) Colon histopathology score. (E) Periodic acid‐Schiff (PAS) stained testis sections. Effect of NJ241 on thickness of seminiferous epithelium (F), and Johnsen's score (G). Results were expressed as mean ± standard deviation (SD) (*n* = 6–8). Parameters marked by the same letter are not significantly different. Significance is represented as *p* < 0.05. BL, low dosage of NJ241; BH, high dosage of NJ241; DSS, dextran sodium sulfate.

A mouse model of colitis was established using a 2% dextran sodium sulfate (DSS) solution to evaluate the effects of NJ241 on UC‐induced reproductive injury. The methodology for establishing the UC model is illustrated in Figure 1A. The induction of colonic tissue inflammation is a critical criterion for successful model development. Histological analysis of hematoxylin and eosin (H&E)‐stained colonic tissue sections was performed, assessing parameters including the severity and extent of inflammation, crypt damage, and focal formation, as described previously [21]. The results revealed that colonic tissue from the control group showed intact structural integrity, whereas the DSS‐treated mice exhibited significant structure damage, characterized by crypt destruction and goblet cell loss. Notably, NJ241 supplementation effectively alleviated DSS‐induced colonic tissue damage, indicated by reduced mucosal crypt injury, improved muscularis mucosa thickness, and decreased infiltration of neutrophils and eosinophils, as well as suppression of crypt granuloma formation (Figure 1B). Colonic histopathology scores confirmed the protective effects of NJ241, demonstrating significant mitigation of ulcerative colitis (Figure 1C). Furthermore, NJ241‐treated mice showed a marked recovery in body weight by day 9 compared to the DSS‐treated controls, with statistically significant differences (*p* < 0.05) (Figure 1D). These findings demonstrate that NJ241 effectively mitigates colonic tissue inflammation and structural damage in UC, restoring colonic integrity and reducing symptoms associated with the disease.

### NJ241 attenuates ulcerative colitis‐induced male reproductive damage

Next, we determined the effect of colitis status on the mouse testis by measuring the epithelial thickness of seminiferous tubules and the Johnsen's scores. Histomorphometric analysis showed a significant reduction in seminiferous tubule thickness in the DSS group, accompanied by prominent luminal vacuolization compared to the other groups (Figure 1E). In contrast, NJ241 supplementation effectively attenuated these changes, restoring testicular morphology, seminiferous tubule thickness, and the Johnsen's scores (Figure 1F,G).

**FIGURE 2:**
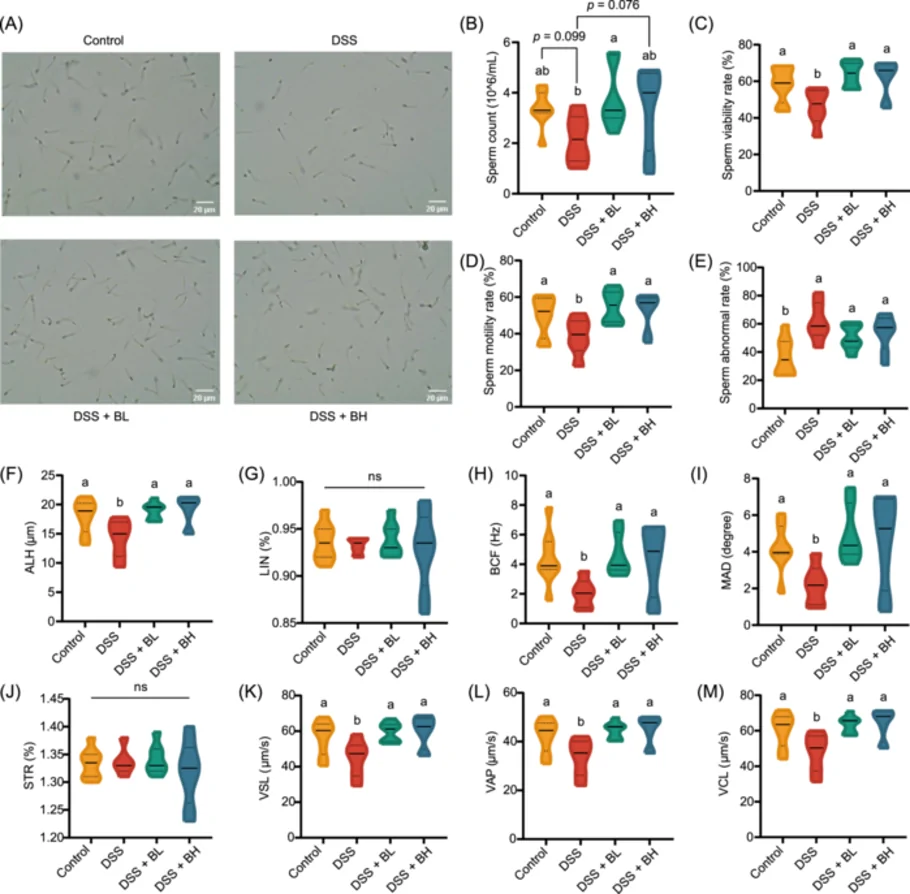
Effects of *Bifidobacterium animalis* subsp. *lactis* NJ241 on sperm quality of UC mice. (A) Representative images of sperm capture. Sperm analysis of sperm count (B), sperm viability (C), sperm motility (D), sperm abnormalities (E), ALH (F), LIN (G), BCF (H), MAD (I), STR (J), VSL (K), VAP (L), VCL (M). Results were expressed as mean ± SD (*n* = 8). Parameters marked by the same letter are not significantly different. Significance is represented as *p* < 0.05. ALH, amplitude of lateral head displacement; BCF, beat cross frequency; BL, low dosage of NJ241; BH, high dosage of NJ241; DSS, dextran sodium sulfate; LIN, linearity; MAD, mean angular displacement; ns, no significance; STR, straightness coefficient; VAP, average path velocity; VCL, curvilinear velocity; VSL, straight‐line velocity.

Sperm quality was further assessed to determine the impact of colitis and the therapeutic effects of NJ241. Parameters, including sperm count, morphology, motility, viability, and abnormality, were analyzed. Results revealed that UC mice exhibited a significant reduction in sperm count and an increased proportion of abnormal spermatozoa (Figure 2A). These observations were further supported by computer‐assisted semen analysis (CASA), which demonstrated a significant decrease in sperm count, viability and motility, together with an increased sperm abnormality rate in UC mice (*p* < 0.05) (Figure 2B–E). Results indicated that NJ241 intervention had no significant effect but modestly reduced the recovery of sperm abnormal rate caused by colitis (Figure 2E). Additionally, colonic inflammation caused significant impairments on other sperm quality parameters, which were effectively reversed by NJ241 treatment (*p* < 0.05) (Figure 2F–M). These results strongly indicate that NJ241 could effectively mitigate the adverse effects of colitis on sperm quality and male reproductive health.

### NJ241 suppresses inflammatory cytokines and enhances intestinal barrier function

**FIGURE 3:**
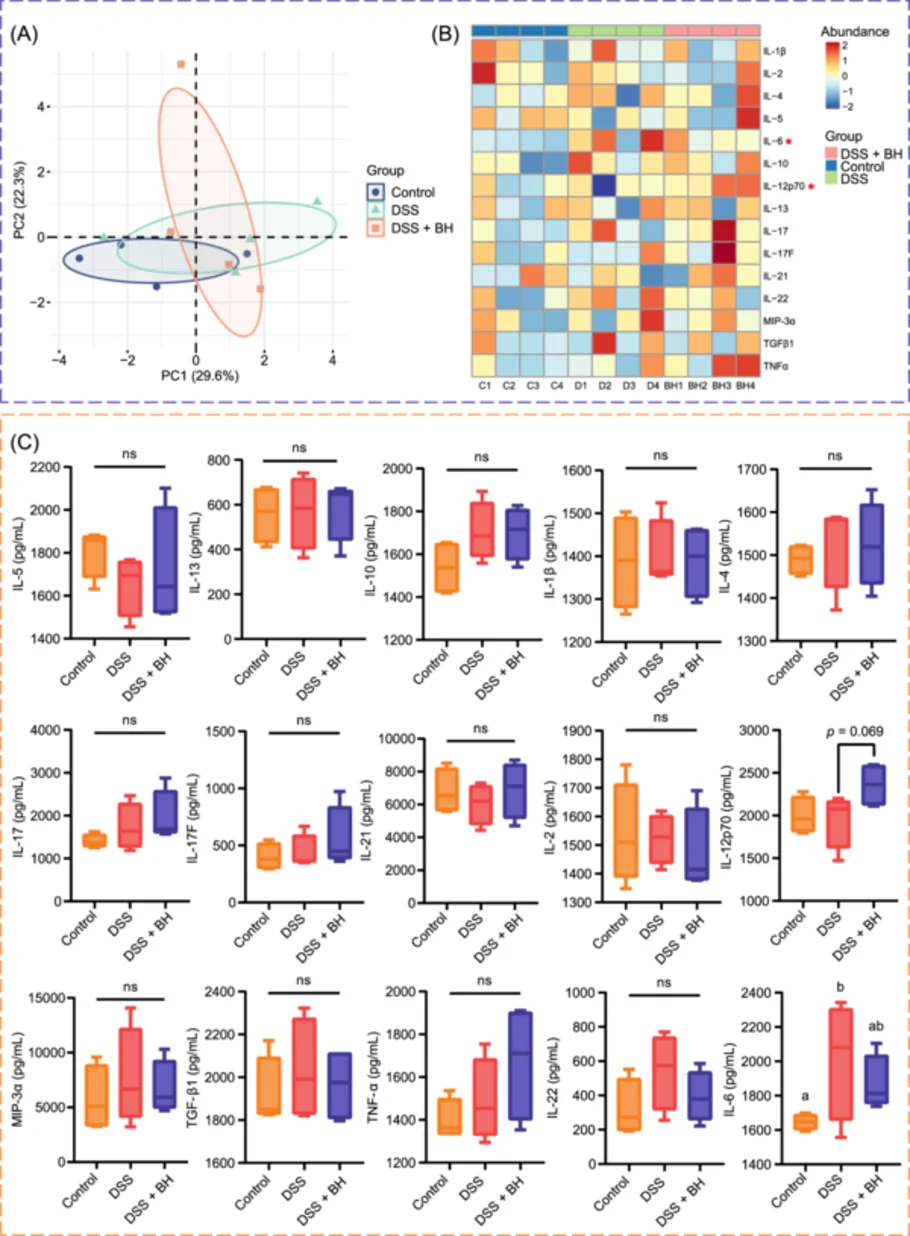
Effects of *Bifidobacterium animalis* subsp. *lactis* NJ241 on cytokine levels in UC mice. (A) Principal component analysis (PCA) plot of cytokines in control group, DSS group, and DSS + BH group. (B) Cluster analysis of cytokines in control group, DSS group, and DSS + BH group. (C) Concentrations of 15 representative inflammatory cytokines. Results were expressed as mean ± SD (*n* = 4). Parameters marked by the same letter are not significantly different. Significance is represented as *p* < 0.05. ns is represented as no significance.

**FIGURE 4:**
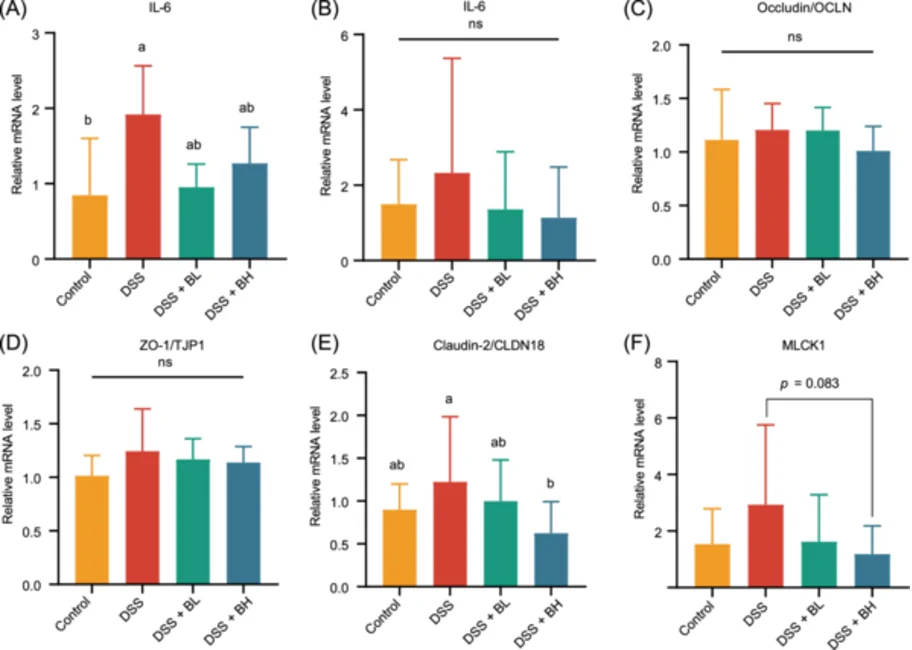
Effects of *Bifidobacterium animalis* subsp. *lactis* NJ241 on the mRNA expression levels of pro‐inflammatory cytokine and tight junction associated genes in testicular tissue and colonic tissue. The relative mRNA expression level of IL‐6 in testicular tissue (A), and the mRNA expression levels of IL‐6 (B), Occludin (C), ZO‐1 (D), Claudin‐2 (E), and MLCK1 (F) in colonic tissue. Results were expressed as mean ± SD (*n* = 4–8). Parameters marked by the same letter are not significantly different. Significance is represented as *p* < 0.05. ns is represented as no significance.

To assess the immune responses in colitis mice, serum levels of multi‐inflammatory cytokines were analyzed. Principal component analysis (PCA) and cluster analysis of the cytokine data showed that the inflammatory profile in the DSS group diverged significantly from the control group, while high‐dose NJ241‐treated group (DSS + BH) closely resembled the control group (Figure 3A,B). Moreover, inflammatory cytokine profiling of the DSS group was clearly distinguished from the other two groups by cluster analysis. It suggests that a high dosage of NJ241 administration may effectively inhibit the inflammatory cytokines, similar to the control group (Figure 3B). Although the levels of most cytokines, including IL‐5, IL‐13, IL‐10, IL‐1*β*, IL‐4, IL‐17, IL‐17F, IL‐21, IL‐2, IL‐12p70, MIP‐3*α*, TGF‐*β*1, TNF‐*α* and IL‐22, showed no statistically significant differences among groups (Figure 3C), IL‐6 production in serum was significantly reduced in the high‐dose NJ241‐treated group compared to the DSS group. Interestingly, further analysis found that mRNA expression levels of IL‐6 in testicular tissue were also significantly inhibited in the high‐dose NJ241‐treated group compared to the DSS group (*p* < 0.05) (Figure 4A). While mRNA expression levels of IL‐6 in colonic tissue exhibited the same trend as IL‐6 production in the serum and testicular tissue (Figure 4B).

The mRNA expression levels of tight junctions (TJs) in colonic tissue, including Occludin, ZO‐1, Claudin‐2, and myosin light chain kinase splice variant 1 (MLCK1), were further assessed. Expression levels of Occludin and ZO‐1 were comparable across the control, DSS, and NJ241‐treated groups (Figure 4C,D). However, NJ241 treatment significantly inhibited the expression levels of Claudin‐2 and MLCK1 compared to the DSS group (Figure 4E,F). These findings suggested that NJ241 may effectively repair gut barrier damage by downregulating the expression level of Claudin‐2 and MLCK1, resulting in reducing gut permeability.

Taken together, the treatment with NJ241 reduces systemic and localized inflammation, particularly through suppression of IL‐6 production, and strengthens intestinal barrier function by regulating TJ‐related protein expression.

### NJ241 reshapes gut microbiota diversity and composition

**FIGURE 5:**
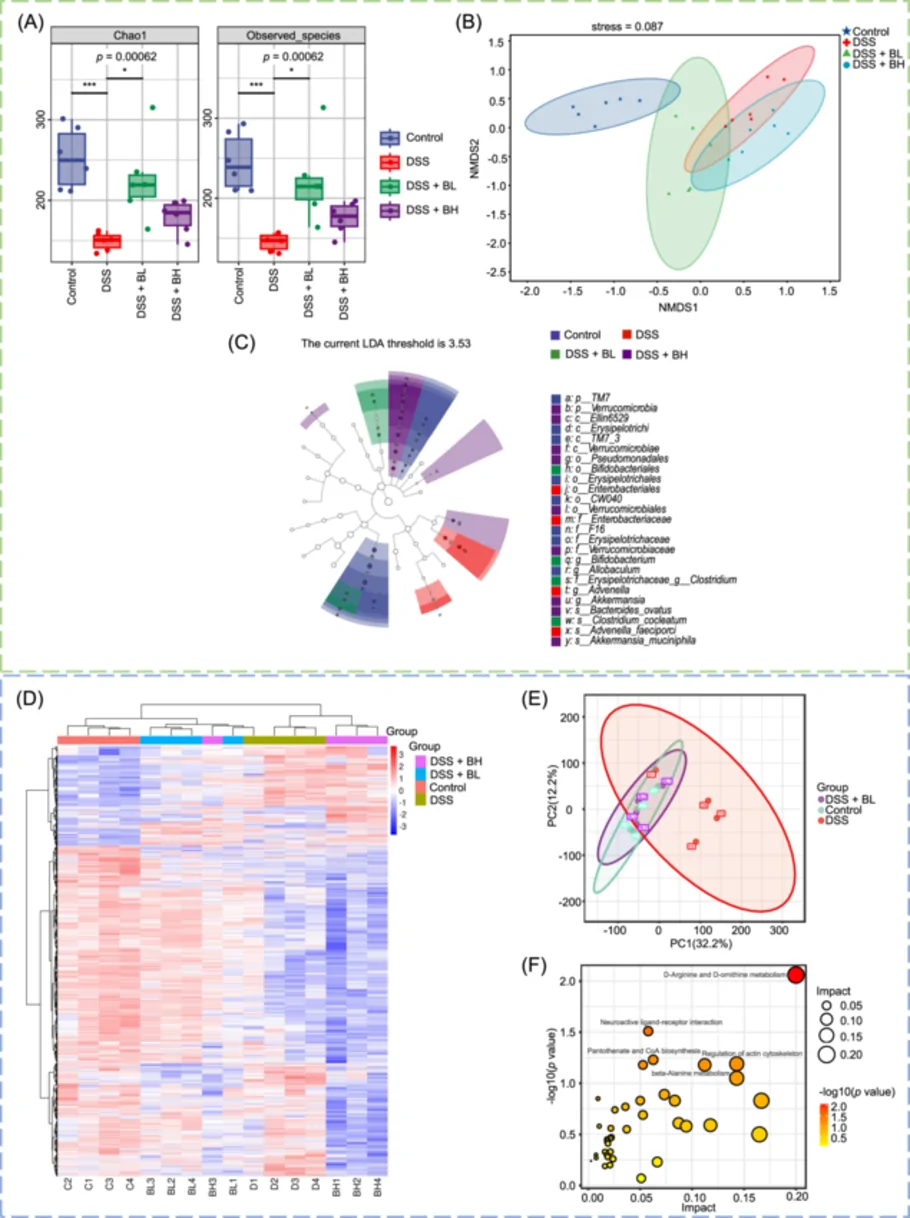
*Bifidobacterium animalis* subsp. *lactis* NJ241 affected the composition of gut microbiota and regulated gut metabolism. (A) Alpha diversity analysis. (B) Nonmetric multidimensional scaling (NMDS) analysis of each group. (C) LEfSe analysis of gut microbiota in all groups. (D) Heat map reflects the differential metabolites between all groups. Red color is represented as high level; blue color is represented as low level. (E) PCA plots analysis of control group, DSS group, and DSS + BL group. (F) Bubble plot of Kyoto Encyclopedia of Genes and Genomes (KEGG) pathway enrichment analysis of differential metabolites among control group, DSS group, and DSS + BL group.

Dysbiosis is a hallmark of UC, and the state of the intestinal microbiota has been shown to influence male reproductive health [22, 23]. Here, we assessed the diversity (*α*‐diversity) of the gut microbiota using the Chao 1 index and the observed species index, both of which are positively correlated with species richness in the samples. As shown in Figure 5A, the Chao 1 and observed species index indicated that the diversity of gut microbiota was significantly reduced following DSS induction (*p* < 0.001). However, the diversity increased significantly after NJ241 intervention (*p* < 0.05), suggesting that NJ241 may improve intestinal flora diversity and potentially attenuate male reproductive injury.

The similarity (*β*‐diversity) of gut microbiota was evaluated using nonmetric multidimensional scaling (NMDS). NMDS analysis illustrates the differences in microbial community composition, with short distances indicating higher similarity [24]. As shown in Figure 5B, NMDS analysis clearly distinguished the control, DSS, DSS + BL, and DSS + BH groups, indicating differences in the microbial community compositions. Notably, the control and low‐dose NJ241‐treated groups (DSS + BL) exhibited a greater similarity in the microbial community compositions. Furthermore, LEfSe analysis identified distinct phylotypes across groups (Figure 5C). At the species level, *Clostridium cocleatum* was enriched in the control group, whereas *Advenella faeciporci* was the most abundant bacterium in the DSS group. In the high‐dose NJ241‐treated group (DSS + BH), *Bacteroides ovatus* and *Akkermansia muciniphila* were identified as the most abundant species. Notably, *Pseudomonadales* exhibited a strong association with high‐dose NJ241 treatment at the order level, which may be closely related to spermatogenesis (Figure 5C).

These results indicate that NJ241 could modulate the diversity and beneficial composition of gut microbiota disrupted by UC, indicating its role in restoring a healthy microbial ecosystem.

### NJ241 modulates gut microbiota metabolism

Clustering analysis of the differential metabolites showed a high degree of similarity between the BL and control groups, which was consistent with the microbial community composition results (Figure 5D). Based on this similarity, the BL group data were used for differential metabolite analysis. PCA of the differential metabolites confirmed that the BL and control groups exhibited higher similarity compared to the DSS group, in agreement with the microbial community composition results (Figure 5E). Additionally, KEGG analysis revealed that the differential metabolites were predominantly enriched in the d‐arginine and d‐ornithine metabolism pathway (Figure 5F). Some studies have also noted that d‐arginine and d‐ornithine metabolism treatments could reduce the inflammatory response to UC [25]. Additionally, an alteration in this pathway will be implicated in reproductive damage in males [26]. Therefore, by influencing metabolic pathways, such as d‐arginine and d‐ornithine metabolism, NJ241 may reduce UC‐associated inflammation and supports improved sperm quality.

### Multi‐omics analysis reveals NJ241 against ulcerative colitis‐stimulated reproductive disorders

**FIGURE 6:**
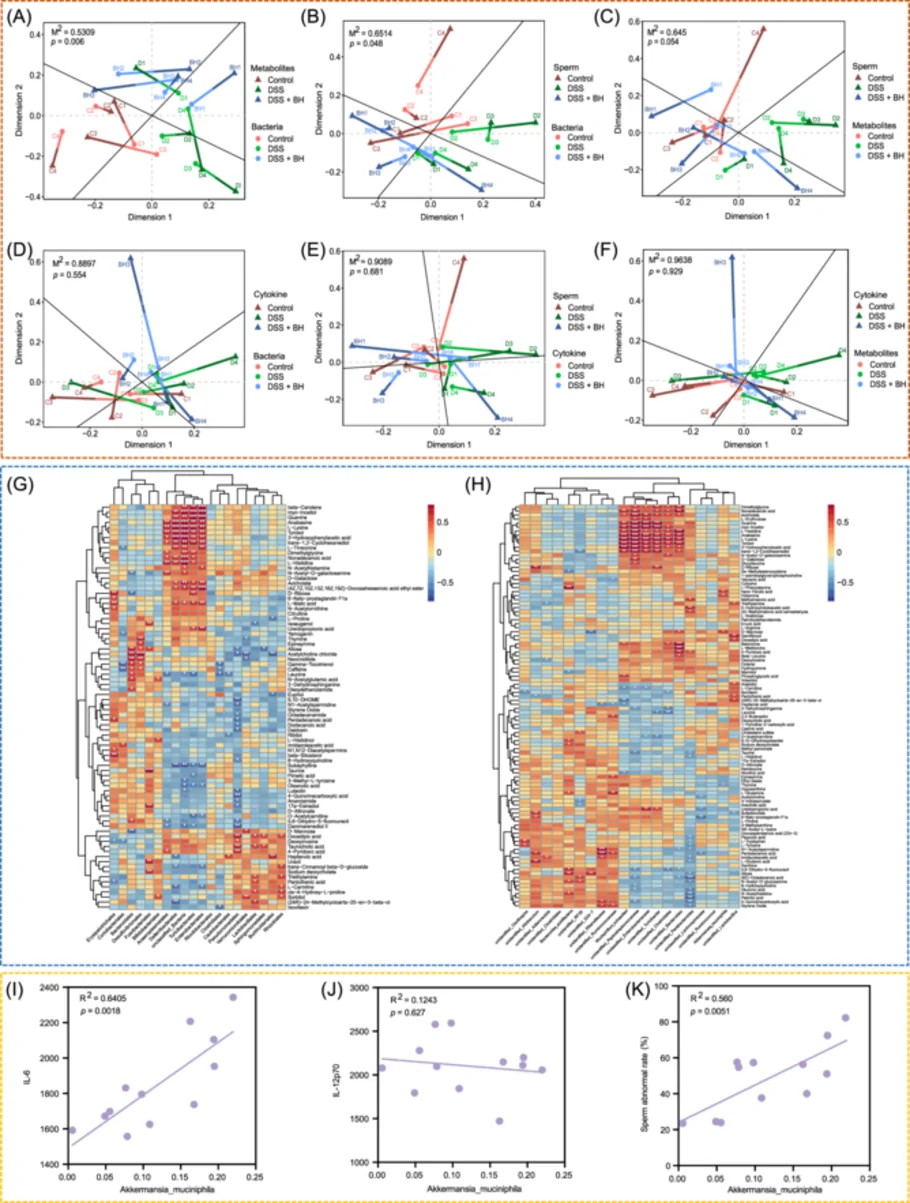
Discovery of interacted pathway of *Bifidobacterium animalis* subsp. *lactis* NJ241 in modulating ulcerative colitis stimulated reproductive disorders through the correlation analysis. (A–F) Procrusters analysis of the associations among gut microbiota (Top 10), metabolites (Top 100), sperm quality, and inflammatory cytokines. Heat map showing gut microbes associated with differential metabolites at order level (G) and species level (H). Red color indicates high levels; blue color indicates low levels. *p < 0.05, **p < 0.01, and ***p < 0.001. Spearman correlation analysis between *Akkermansia muciniphila* and IL‐6 (I), IL‐12p70 (J), and sperm abnormal rate (K).

Procrustes analysis was conducted to assess the associations among gut microbiota (Top 10), metabolites (Top 100), sperm quality, and inflammatory cytokines. As shown in Figure 6A,B, significant correlations were identified between gut microbiota and metabolites (*p* < 0.01), as well as between gut microbiota and sperm quality (*p* < 0.05). Meanwhile, metabolites exhibited a marginal correlation with sperm quality with slight significance (*p* = 0.054) (Figure 6C). Additionally, the correlations between inflammatory cytokine, gut microbiota, metabolites, and sperm quality were analyzed as well, but no significant correlations were found (Figure 6D–F).

A conjoined analysis of gut microbiota and differential metabolites revealed that bacteria taxa such as *Enterobacteriales*, *Turicibacterales*, *Deferribacterales*, and *Anaeroplasmatales* positively correlated with metabolites including anabasine, *L*‐lysine, tyrosol, 3‐hydroxyphenylacetic acid, and trans‐1, 2‐cyclohexanediol. In contrast, *Pseudomonadales* was negatively correlated with metabolites such as dammarenediol II, 4‐quinolinecarboxylic acid, and 9,10‐DHOME, while showing positive correlations with oxoadipic acid, deoxyinosine, taurocholic acid, and 4‐pyridoxic acid (Figure 6G).

At the species level, *Bacteroides_acidifaciens*, *Mucispirillum_schaedleri*, and *Akkermansia muciniphila* were identified as key gut bacteria correlated to the metabolites (Figure 6H). *Bacteroides_acidifaciens* displayed strong correlations with several metabolites, including *L*−Phenylalanine (*p* < 0.001). *Mucispirillum schaedleri* showed significant correlations with metabolites such as Anabasine, *L*−Lysine, Tyrosol, and 3−Hydroxyphenylacetic acid, while *Akkermansia muciniphila* was associated with trans‐ferulic acid, histamine, d‐mannose, and Thymine (Figure 6H). These results were consistent with the LEfSE analysis, suggesting that *Akkermansia muciniphila* could be a biomarker pivotal for the molecular mechanism by which NJ241 mitigates colitis‐induced reproductive disorders. To further explore this, spearman correlation was performed to examine associations between *Akkermansia muciniphila*, sperm quality, and inflammatory cytokines. As shown in Figure 6I–K, *Akkermansia muciniphila* was strongly positively correlated with the inflammatory cytokine IL‐6 (*p* < 0.01, *R* ^2^ > 0.6) and sperm abnormal rate (*p* < 0.01).

Collectively, comprehensive multi‐omics analysis reveals that NJ241 alleviates colitis‐induced reproductive dysfunction by modulating gut bacteria, particularly Akkermansia muciniphila, and its associated metabolites, offering insights into its therapeutic mechanisms.

## 3 DISCUSSION

UC is strongly associated with gut inflammation and microbial ecological dysregulation. Numerous studies have emphasized the pivotal role of the gut microbiota and its metabolites in host physiological processes, including immune, metabolic, neural, and nutritional homeostasis [27, 28]. Our previous study demonstrated that UC can adversely affect male reproductive function [29]. Probiotics have emerged as a promising approach for alleviating UC due to their ability to improve the intestinal epithelial barrier and modulate inflammatory responses, with minimal side effects and significant health benefits [30, 31].

This study aimed to investigate the effects of NJ241 on alleviating reproductive damage in males with colitis, as well as to explore the potential mechanisms through which gut microbiota metabolites contribute to UC and testicular protection. As shown in Figure 1, NJ241 significantly attenuated weight loss and improved colonic histopathology by reducing intestinal inflammatory infiltration and restoring intestinal structural integrity, effectively alleviating colitis.

Male fertility assessment typically relies on semen analysis, the standards for accurately predicting reproduction potential [32]. Through testicular histopathology and semen analysis, we reaffirmed the adverse effects of UC on male reproductive function. Notably, NJ241 demonstrated remarkable to improve sperm quality in the current study. Previous studies have shown that probiotic strains such as *Lactobacillus rhamnosus* CECT8361 and *Bifidobacterium longum* CECT7347 could effectively improve sperm motility while reducing DNA fragmentation and reactive oxygen species (ROS) levels in asthenozoospermic mice [33]. However, it remains unknown as to whether UC‐induced reproductive damage can be alleviated by modulating gut microbiota.

A hallmark pathological feature of UC is persistent colonic inflammation, characterized by the recruitment of diverse inflammatory cells that produce dysregulated cytokines involved in the pathology of UC [34]. While UC has traditionally been considered a T helper (Th) 2‐mediated disease, emerging evidence suggests that cytokine levels vary depending on the disease stage [35]. During the onset stage, cytokines such as TNF‐*α*, IL‐1*β*, and IL‐6, predominantly produced by innate immune cells, are particularly abundant [34]. In the present study, we examined the serum cytokine levels in colitis mice and found that only IL‐6 was significantly elevated. This aligns with the possibility that many cytokines are restricted to the tissue site of inflammation, have relatively short half‐lives, and may only appear in circulation during advanced disease stages [36]. Additionally, IL‐6 and its associated cytokine family play essential roles in maintaining metabolic homeostasis and are implicated in the pathophysiology of a wide range of gastrointestinal and hepatic disorders, which makes them attractive therapeutic targets [37]. In this work, we analyzed the mRNA expression levels of IL‐6 in the testis and colon of UC mice and discovered that the relative expression levels of IL‐6 were significantly elevated in both tissues. This suggests that persistent colonic inflammation may influence testicular function through systemic circulation, thereby exacerbating reproductive impairment associated with colitis. Overexpression of IL6 is known to disrupt blood‐testis barrier (BTB) integrity, disrupt the localization and homeostasis of BTB integral membrane proteins, and ultimately impair spermatogenesis [38]. Although IL6 mRNA expression levels in the colon did not significantly differ between groups, the observed trend mirrored the serum analysis results, reinforcing the systemic impact of colonic inflammation on reproductive health.

The intestinal epithelial TJ barrier controls paracellular permeability, controlling the transportation of luminal contents into intestinal tissues and systemic circulation [39]. To further elucidate the connection between intestinal inflammation and reproductive injury, we examined colonic mRNA expression of TJ‐related proteins. Interestingly, unlike previous studies, the mRNA levels of Occludin and ZO‐1 showed no significant difference among groups [40, 41]. However, the relative mRNA levels of Claudin‐2 tended to increase in the colitis mice, while NJ241 intervention modestly reduced Claudin‐2 levels. Claudin‐2, predominantly expressed in the TJ region and the apical cytoplasm of surface colonocytes, has been implicated in the regulation of paracellular permeability by forming cation‐ and water‐selective channels in the intestinal epithelium [42, 43]. Claudin‐2 upregulation was initially described in IBD, including UC and CD. Notably, Claudin‐2 overexpression has been associated with enhanced resistance to experimental colitis by the suppression of the IL‐6‐induced NF‐κB pathway [44], which may partly explain the lack of significant changes in IL‐6 mRNA levels in the colon observed in the current study. Additionally, the permeability of the TJ leak pathway can be regulated by MLCK1, a key effector and potential therapeutic target in barrier dysfunction [45]. NJ241 treatment reduced mRNA levels of Claudin‐2 and MLCK1, implying a protective role in preserving intestinal epithelial barrier function. Wu et al. [46] also suggested that probiotic administration could improve mechanical barrier by decreasing Claudin‐2 levels in UC mice. When intestinal permeability increases, harmful substances enter the bloodstream, putting the immune system on high alert and leading to systemic inflammation, which may also lead to decreased sperm quality.

Gut microbiota plays an important role in the testis, modulating the permeability of the BTB and contributing to the regulation of endocrine function in the testis [13]. To investigate the differences in gut microbiota among groups, we employed both α‐diversity analysis and β‐diversity analyses. Consistent with the findings by Tong et al. [16], the gut microbiota profiles of the control and DSS groups exhibited clear separation, indicating significant microbial dysbiosis in the colitis model. In this study, LEfSe analysis of intestinal flora was employed to identify potential biomarkers. We found that *Clostridium cocleatum* was enriched in controls. However, some studies have shown that the abundance of *Clostridium cocleatum* is elevated in colorectal cancer models, therefore the link between *Clostridium cocleatum* and colitis is not clear [47]. Additionally, *Advenella faeciporci*, was found enriched in UC mice, but its specific association with colitis and reproductive function has not been elucidated. In contrast, *Bacteroides ovatus* and *Akkermansia muciniphila*, enriched in the BH group, have been linked to beneficial health effects. Reduced abundance of these bacteria is associated with obesity, diabetes, and inflammation [48, 49]. We hypothesize that NJ241 treatment suppresses intestinal inflammation by increasing the abundance of *Bacteroides ovatus* and *Akkermansia muciniphila*. Furthermore, fecal virome transfer has been shown to stimulate *Akkermansia muciniphila* proliferation, which may enhance male fertility [50]. Interestingly, *Pseudomonadales* also showed significant enrichment in the BH group. The abundance of *Pseudomonas* is directly associated with total motile sperm count [51]. Han et al. [19] demonstrated a positive correlation between reduced *Pseudomonas* abundance in men with asthenozoospermia and the core differential metabolite hexadecanamide. These findings suggest that NJ241 effectively modulates gut microbiota to improve sperm motility.

It is well‐known that metabolic regulation is crucial for spermatogenesis [25]. The gut microbiota is involved in the regulation of multiple host metabolic pathways, which not only function in metabolism‐related diseases but also affect other systems, such as the reproductive system [26]. Our study showed that the differential metabolites in the low‐dose NJ241‐treated group exhibited greater similarity to the control group. KEGG pathway analysis revealed that these differential metabolites were predominantly enriched in the d‐arginine and d‐ornithine metabolism pathway. Abnormalities in amino acid metabolism in the colonic tissues have been observed in UC patients [52]. d‐arginine and d‐ornithine metabolism treatments reduce the inflammatory response to UC [53]. Furthermore, alterations in l‐arginine in the metabolic pathway of d‐arginine and d‐ornithine may explain the positive effects of anthocyanin in attenuating busulfan‐induced reproductive damage in males [26]. Therefore, NJ241 may regulate the homeostasis of gut microbiota and reduce the level of inflammation through targeted intervention of d‐arginine and d‐ornithine metabolism, which in turn improves sperm quality. However, the specific molecular mechanisms underlying this regulation warrant further investigation.

One of the main modes of gut microbiota‐host interaction is through metabolites, and many studies have described alterations in the composition of the gut microbiota and metabolite profiles in IBD patients [54]. In this study, we conducted correlation analyses to explore associations between gut microbiota and differential metabolites at the order level. Metabolites such as dammarenediol II, 4‐quinolinecarboxylic acid, and 9,10‐DHOME were reduced, whereas metabolites such as oxoadipic acid, deoxyinosine, taurocholic acid, and 4‐pyridoxic acid were significantly increased in NJ241‐treated mice compared to the UC mice.

9,10‐DHOME is a ligand for PPAR*γ*, which regulates fatty acid metabolism and inflammation‐related genes expression. PPAR*γ* activation inhibits inflammation‐responsive transcription factors NF‐*κ*B and AP‐1 [55]. However, the precise mechanism by which 9,10‐DHOME restores male reproductive function remains unclear. Oxoadipic acid has been shown negatively correlated with sperm abnormalities [56], and its levels are lower in the gut of aging mice with spermatogenic dysfunction [57]. Thus, the increased oxoadipic acid levels observed in NJ241‐treated mice may contribute to improved sperm quality. Additionally, we found that 3‐hydroxyphenylacetic acid (3‐HPAA) was positively correlated with the abundance of *Enterobacteriales*, *Turicibacterales*, *Anaeroplasmatales*, and other microbes. Previous studies suggest that 3‐HPAA possesses antioxidant and antiapoptotic activities, enhancing the expression of GPX4 and NRF2, which suggests a role in alleviating spermatogenic dysfunction by regulating ferroptosis [57]. At the species level, *Bacteroides_acidifaciens*, *Mucispirillum_schaedleri*, and *Akkermansia muciniphila* were found to be significantly correlated with 3‐HPAA. LEfSE analysis further indicated that *Akkermansia muciniphila* was the crucial biomarker associated with the effects of NJ241 in managing colitis‐induced reproductive disorders. Specifically, *Akkermansia muciniphila* was significantly correlated with trans‐ferulic acid, histamine, d‐mannose, and Thymine. Trans‐ferulic acid has been linked to UC amelioration and improved sperm quality. It can be produced by intestinal microorganisms and cross the colonic barrier, which exerts antioxidant and anti‐inflammatory effects by regulating TLR4‐/IRF3, JAK1/STAT‐3/ERK1/2, MAPK/NF‐*κ*B‐p65, and *p*‐AKT/AKT signaling [58, 59]. Further spearman correlation analysis also indicated that *Akkermansia muciniphila* was positively correlated with hematologic cytokine IL‐6 and sperm abnormality rate. This suggests that *Akkermansia muciniphila* may promote the effects of trans‐ferulic acid, reducing the secretion of inflammatory cytokines, which subsequently reduces systemic inflammation and improves sperm quality.

Collectively, the integrated multi‐omics and multi‐cytokine profile data suggest that NJ241 may significantly ameliorate colitis‐induced reproductive disorders by modulating *Akkermansia muciniphila* and its metabolite trans‐ferulic acid. These findings elucidate potential molecular pathways by which NJ241 could be used to modulate gut microbiota to improve reproductive health.

## 4 CONCLUSION

**FIGURE 7:**
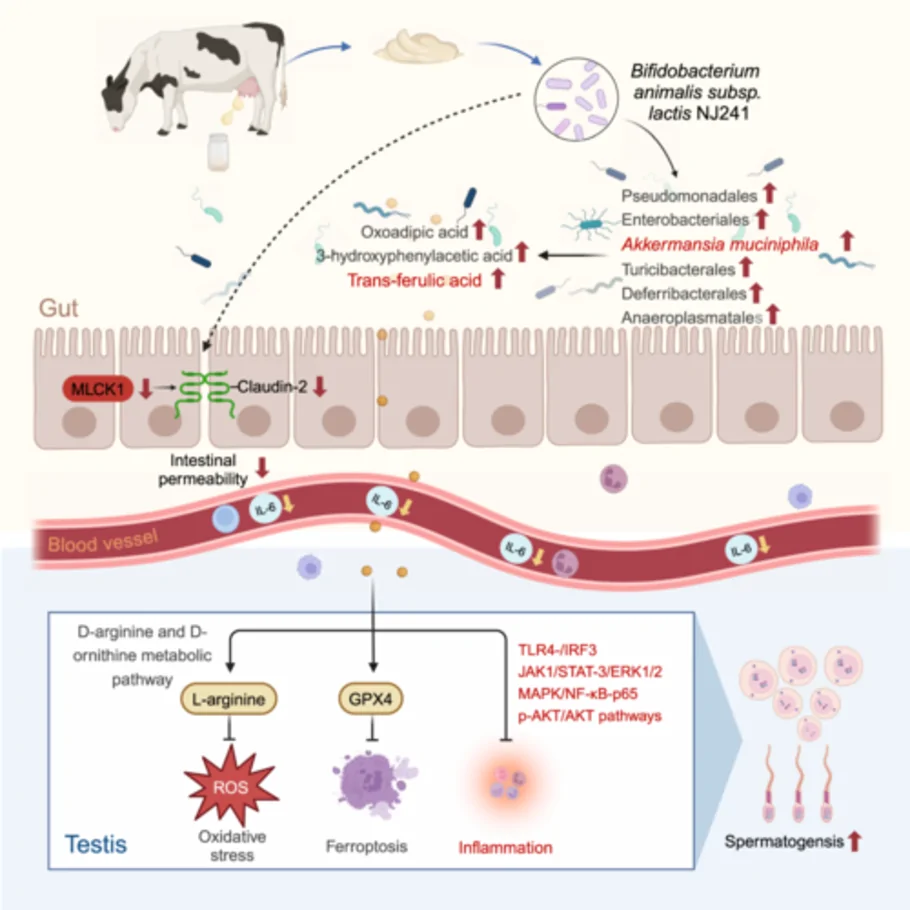
Schematic illustration of the potential molecular mechanism of probiotic *Bifidobacterium animalis* subsp. *lactis* NJ241 in managing colitis‐stimulated reproductive disorders. *Bifidobacterium animalis* subsp. *lactis* NJ241 could be isolated from naturally fermented bovine milk. Dietary supply with NJ241 may effectively improve testicular spermatogenesis through the inhibition of oxidative stress, ferroptosis, and inflammation‐associated pathways, mediated through increasing the abundance of *Akkermansia muciniphila*‐linked metabolite trans‐ferulic acid to down‐regulate the expression levels of MLCK1 and Claudin‐2, thereby leading to a decrease in intestinal permeability and the expression levels of pro‐inflammatory cytokine IL‐6.

Milk is the natural nutraceutical containing numerous functional ingredients. *Bifidobacterium animalis* subsp*. lactis* NJ241, a potential probiotic isolated from naturally fermented bovine milk, remains poorly characterized. This study aimed to investigate the therapeutic effects and underlying molecular mechanisms of NJ241 in alleviating colitis and its associated reproductive disorders by analyzing gut microbiota, metabolome, and multi‐cytokines profiling. Our results demonstrated that NJ241 could effectively alleviate colitis symptoms and improve sperm quality. Additionally, NJ241 treatment restored the expression levels of Claudin‐2 and MLCK1, reducing intestinal permeability. Notably, NJ241 significantly inhibited the expression level of the pro‐inflammatory cytokine IL‐6, regulating intestinal inflammation and metabolic homeostasis. LEfSe analysis of gut microbiota (Figure 5C) also identified the enrichment of *Pseudomonadales* and *Akkermansia muciniphila* in the NJ241‐treated group, which are linked to spermatogenesis and inflammation remission. Metabolomic analysis revealed that gut metabolites were enriched in the *D*‐arginine and *D*‐ornithine metabolic pathways, processes closely associated with oxidative stress regulation during spermatogenesis, suggesting a potential mechanism by which NJ241 enhances sperm quality [26]. Conjoint analysis of gut microbiota and metabolomics revealed that oxoadipic acid, 3‐hydroxyphenylacetic acid, and trans‐ferulic acid may be identified as the metabolite markers influenced by NJ241 treatment. These metabolites were significantly associated with bacterial taxa such as *Pseudomonadales*, *Enterobacteriales*, *Turicibacterales*, *Deferribacterales*, *Anaeroplasmatales*, and *Akkermansia muciniphila* (Figure 7). Further analysis indicated that NJ241 might improve testicular spermatogenesis through inhibiting TLR4 signaling, mediated through modulation of *Akkermansia muciniphila* and its metabolite trans‐ferulic acid. These findings highlight the potential of NJ241 as a probiotic derived from natural foods, positioning it as a promising candidate for applications in nutritional healthcare. Additionally, this study provides corroborative evidence for the reproductive health benefits of naturally fermented bovine milk.

## 5 METHODS

### NJ241 preparation and purification

*Bifidobacterium animalis* subsp*. lactis* NJ241 strain (CGMCC No.20816) was isolated from naturally fermented milk. Fermented milk samples were collected and diluted for further cultivation on MRS solid medium under anaerobic conditions at 37°C to obtain single colonies. *Bifidobacterium animalis* subsp*. lactis* NJ241 was obtained by genomic DNA identification and analysis and was preserved in the China Microbial Strain Preservation Center.

### Materials and reagents

DSS (molecular weight: 36–50 kDa) was purchased from MP Biomedicals. 4% formalin was purchased from Servicebio Biotechnology Co., Ltd. Bouin fixative solution, H&E, and periodic acid‐Schiff (PAS) reagents were purchased from Biossci Biotechnology Co., Ltd. ProcartaPlex^TM^ immunoassay kit for the determination of cytokines was obtained from Invitrogen. Primers were obtained from Tsingke Biotech Co., Ltd. PrimeScript^TM^ RT reagent kit was obtained from Takara Bio Inc.

### Animal's model

C57BL/6JNidfc mice (male, 42–55 days, 20 ± 1 g) were obtained from Guangdong Vital River Laboratory Animal Technology Co., Ltd. Animals were adapted to the laboratory environment with a relative humidity of 40%–70%, a standard 12 h light/dark cycle, and a temperature of 22 ± 1°C for 7 days before the experiment. The animals were allowed free access to standard chow and sterilized water.

UC model was established by administration of 2% (w/v) DSS solution as described previously with minor modifications Chen et al. [60]. Briefly, 32 mice were randomly divided into 4 groups: the control group (control), the DSS‐treated group (DSS), the low‐dose NJ241 group (DSS + BL, 5 × 10^8^ CFU/mL), and the high‐dose NJ241 group (DSS + BH, 5 × 10^9^ CFU/mL). To induce UC in the DSS‐treated groups (DSS, BL, and BH), drinking water was replaced with 2% DSS solution for 7 days. Subsequently, all cages were switched back to distilled water for 2 days. The mice in the control and DSS groups were received daily oral gavage with 200 μL of distilled water, while the low‐dose NJ241 group and the high‐dose NJ241 group were received daily oral gavage 200 μL of NJ241. Body weight was monitored daily. On day 9, all mice were euthanized under anesthesia. Colonic tissues were fixed with 4% paraformaldehyde, testicular tissues were fixed with Bouin fixative solution, and fecal samples were stored at −80°C.

### Histopathological evaluation

After fixation, 4 μm sections of colon and testicular tissues were prepared. These sections were stained with H&E and PAS. Histopathological evaluation was then performed using an Optec BK‐FL microscopy (Chongqing Optec Instrument Co., Ltd). Colon histopathology scoring was guided by the method described by Chen et al. [21]. Histopathological analysis of the testis included measuring the thickness of seminiferous tubules and quantifying the Johnsen's score of spermatogenesis. Johnsen's score was assessed based on the morphology and number of germ cells at different stages of spermatogenesis, following the criteria outlined by Johnsen [61].

### Sperm quality analysis

Sperm quality analysis was performed according to a previously published method [29]. Briefly, epididymal spermatozoa were collected from the left caudal epididymis. The caudal epididymis was quickly removed post‐euthanasia, placed in saline solution, and incised with micro‐scissors. The tissue was then incubated at 37°C for 5 min to allow spermatozoa release. The resulting sperm samples were then transferred to a preheated counting chamber and analyzed using the CASA system. At least 30 fields were assessed for each sample.

### Inflammatory cytokines measurement

Blood samples were collected post‐euthanasia and centrifuged at 3500 *g* for 15 min at 4°C. Then the serum was collected and stored at −80°C until further analysis. Serum cytokine levels were analyzed using an immunoassay kit according to the manufacturer's instructions.

### Quantitative real‐time PCR analysis

RNA was extracted from colonic and testicular tissues using from TRIzol reagent. cDNA synthesis was then synthesized using the PrimeScript^TM^ RT reagent kit. Gene expression was analyzed by quantitative real‐time PCR (qRT‐PCR) using a Bio‐rad Real‐Time PCR system. GAPDH was used as a reference gene to normalize gene expression. The primer sequences are listed in Table S1.

### 16S rRNA sequencing

16S rRNA gene V3‐V4 regions were amplified using universal primers, 806R and 515F. PCR products were visualized on an agarose gel, and bands of approximately 400–450 bp were purified using a Qiagen Gel Extraction Kit. Purified PCR products were sequenced on the Illumina Novaseq. 6000 platform. High‐quality sequence reads were processed using QIIME (version 1.7.0). Sequences with ≥97% similarity were clustered into operational taxonomic units.

### Nontargeted metabolomics analysis

Nontargeted metabolomics analysis was performed using liquid chromatography‐mass spectrometry (LC‐MS). Briefly, thawed samples were vortexed, and 500 μL methanol was added for metabolite extraction. The mixture was then centrifuged at 12,000 rpm for 10 min at 4°C, and the entire supernatant was collected for further analysis. The supernatant was subsequently collected for nontargeted metabolomics analysis. The LC analysis was performed on a Vanquish UHPLC System (Thermo Fisher Scientific) equipped with an ACQUITY UPLC® HSS T3 column (150 × 2.1 mm, 1.8 μm) (Waters). Sample acquisition and analysis followed the manufacturer's instructions for the LC‐MS system. Orthogonal Projections to Latent Structures Discriminant Analysis were applied to remove irrelevant variables. Metabolites with a variable importance in projection were set as >1 and *p*‐value < 0.05 were considered significant and subsequently mapped to the pathways in the Kyoto Encyclopedia of Genes and Genomes (KEGG) database (http://www.kegg.jp/kegg/pathway.html).

### Multi‐omics data integration

Procrustes analysis was employed to assess the significant associations among the top 10 gut microbes, top 100 metabolites, sperm quality, and inflammatory cytokines. Next, spearman correlation analysis was used to examine the relationships between metabolites and bacteria. All the analyses and visualizations were performed with R software (Version 4.0.2) and the OmicStudio tools at https://www.omicstudio.cn/tool.

### Statistical analysis

All data were normally distributed and are presented as mean ± standard deviation (SD). Statistical analyses were performed using GraphPad Prism 8.0 and SPSS version 26.0 (SPSS Inc.). A one‐way analysis of variance with Tukey's least significant difference post hoc text was used to determine statistical significance (*p* < 0.05) across multiple groups.

## AUTHOR CONTRIBUTIONS

**Jingmin Lin**: Writing—review and editing; data curation; visualization; software. **Lingzi Yin**: investigation; writing—original draft; formal analysis; data curation; visualization; software. **Yueyao Fan**: Writing—review and editing; formal analysis. **Zhiling Yu**: resources; writing—review and editing. **Xin Ma**: Conceptualization; resources; funding acquisition; writing—review and editing. **Xiuqiong Fu**: Writing—review and editing. **Yong Zhang**: Writing—review and editing. **Shaojun Tang**: Resources; supervision; writing—review and editing; project administration. **Jiali Chen**: Conceptualization; methodology; data curation; investigation; project administration; funding acquisition; writing—original draft; writing—review and editing; resources; supervision; visualization; validation.

## CONFLICT OF INTEREST STATEMENT

The authors declare no conflicts of interest.

## 1 ETHICS STATEMENT

The ethics application (No. IACUC‐20230526‐07) was approved by the Research Ethics Committee of Jinan University.

## Supporting information

TABLE: S1 PCR primers.

## Data Availability

The data that support the findings of this study are available from the corresponding author upon reasonable request. All the raw data used in this research are available in the NCBI SRA data set (BioProject: PRJNA1184710, at: https://www.ncbi.nlm.nih.gov/bioproject/PRJNA1184710/) and the EMBL‐EBI MetaboLights database with study identifier MTBLS11374 at https://www.ebi.ac.uk/metabolights/MTBLS11374. The data and scripts used are saved in https://github.com/kellychan123/CJL.git. Supplementary materials (tables, graphical abstracts, slides, videos, Chinese translated versions, and updated materials) can be found in the online DOI or iMeta Science http://www.imeta.science/imetaomics/.
